# Comparative assessment of the bacterial communities associated with *Anopheles darlingi* immature stages and their breeding sites in the Brazilian Amazon

**DOI:** 10.1186/s13071-023-05749-6

**Published:** 2023-05-01

**Authors:** Katherine D. Mosquera, Louise K. J. Nilsson, Marta Rodrigues de Oliveira, Elerson Matos Rocha, Osvaldo Marinotti, Sebastian Håkansson, Wanderli P. Tadei, Antonia Queiroz Lima de Souza, Olle Terenius

**Affiliations:** 1 Department of Cell and Molecular Biology, Biomedical Centre (BMC), Uppsala University, Uppsala, Sweden; 2 Department of Ecology, Swedish University of Agricultural Sciences (SLU), Uppsala, Sweden; 3 Programa de Pós-graduação em Biodiversidade e Biotecnologia (PPG-BIONORTE), Universidade do Estado do Amazonas, Manaus, Brazil; 4 Department of Entomology and Acarology, Luiz de Queiroz College of Agriculture (ESALQ), University of São Paulo, Piracicaba, Brazil; 5 School of Agricultural Sciences, Department of Bioprocesses and Biotechnology, Central Multi User Laboratory, São Paulo State University (UNESP), Botucatu, Brazil; 6 Department of Biology, Indiana University, Bloomington, IN USA; 7 Division of Applied Microbiology, Department of Chemistry, Lund University, Lund, Sweden; 8 Laboratório de Malária e Dengue, Instituto Nacional de Pesquisas da Amazônia, Manaus, Brazil; 9 Faculdade de Ciências Agrárias, Universidade Federal do Amazonas, Manaus, Brazil

**Keywords:** *Anopheles darlingi*, Microbiota, Breeding sites, Amazon, Malaria

## Abstract

**Background:**

The neotropical anopheline mosquito *Anopheles darlingi* is a major malaria vector in the Americas. Studies on mosquito-associated microbiota have shown that symbiotic bacteria play a major role in host biology. Mosquitoes acquire and transmit microorganisms over their life cycle. Specifically, the microbiota of immature forms is largely acquired from their aquatic environment. Therefore, our study aimed to describe the microbial communities associated with *An. darlingi* immature forms and their breeding sites in the Coari municipality, Brazilian Amazon.

**Methods:**

Larvae, pupae, and breeding water were collected in two different geographical locations. Samples were submitted for DNA extraction and high-throughput *16S rRNA* gene sequencing was conducted. Microbial ecology analyses were performed to explore and compare the bacterial profiles of *An. darlingi* and their aquatic habitats.

**Results:**

We found lower richness and diversity in *An. darlingi* microbiota than in water samples, which suggests that larvae are colonized by a subset of the bacterial community present in their breeding sites. Moreover, the bacterial community composition of the immature mosquitoes and their breeding water differed according to their collection sites, i.e., the microbiota associated with *An. darlingi* reflected that in the aquatic habitats where they developed. The three most abundant bacterial classes across the *An. darlingi* samples were Betaproteobacteria, Clostridia, and Gammaproteobacteria, while across the water samples they were Gammaproteobacteria, Bacilli, and Alphaproteobacteria.

**Conclusions:**

Our findings reinforce the current evidence that the environment strongly shapes the composition and diversity of mosquito microbiota. A better understanding of mosquito–microbe interactions will contribute to identifying microbial candidates impacting host fitness and disease transmission.

**Graphical Abstract:**

**Figure:**
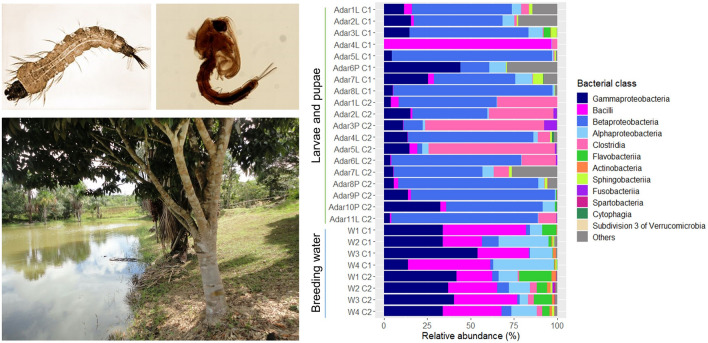

**Supplementary Information:**

The online version contains supplementary material available at 10.1186/s13071-023-05749-6.

## Background

Mosquitoes harbor a variety of microbes, including bacteria, fungi, viruses, and protists [1]. The set of these microorganisms, collectively known as microbiota, are acquired from different sources throughout the host’s life [2, 3]. Aquatic niches, where immature forms develop, have been depicted as a main source of microbial acquisition [4–6]. This is because mosquito breeding water holds a wide diversity of microorganisms on which larvae feed and obtain symbionts [7–9]. These larvae–microorganism interactions have an impact on early-stage development as well as carryover effects on adult fitness [10].

Mosquito microbiota plays a pivotal role in host metabolism, including blood and sugar digestion [11–13], supply of vitamins and amino acids [14], life-history traits such as survival [15], oviposition site choice [16, 17], egg production [12, 18], and vector competence [19, 20]. Thus, mosquito-associated microbes have drawn attention in recent years due to the emerging evidence showing their influence on insect hosts.

*Anopheles darlingi* is the main malaria vector in South America, transmitting *Plasmodium falciparum* and *Plasmodium vivax* in the endemic areas of Amazonian countries [21–23]. Since this mosquito exhibits a high degree of plasticity in its biting behavior, switching from increased exophagy due to repellency to insecticide-treated nets and subsequently reverting to increased endophagy as nets become worn, it is challenging to control using standard methods, i.e., long-lasting insecticidal nets and indoor residual spraying [24]. Moreover, its feeding preference for humans [25, 26] plus its increased attraction to individuals infected with *P. vivax* [27] highlights the role of *An. darlingi* in malaria transmission.

The manipulation of bacteria that colonize mosquitoes has become a promising avenue for the development of novel strategies for controlling the transmission of vector-borne diseases [1, 28]. Several authors have explored the microbiota associated with *An. darlingi* and their breeding sites using both culture-dependent and independent methodologies [29–35]. Some of these studies already suggested paratransgenesis as a method to reduce malaria transmission by this neotropical malaria vector, as bacteria commonly reported as promising candidates, such as *Serratia*, *Pantoea*, and *Asaia*, were frequently reported in association with *An. darlingi* mosquitoes [34–37].

Considering the potential impact of bacteria on vector competence, understanding the factors shaping the community composition of mosquito microbiota, such as developmental stage, environment, and geographical location, could contribute to assessing whether certain mosquito populations are more likely to transmit pathogens than others [38]. This study intends to contribute to the knowledge about the bacteria associated with neotropical anopheline mosquitoes. We used 16S ribosomal RNA (rRNA) amplicon gene sequencing to comparatively assess the bacterial communities in *An. darlingi* larvae and pupae and their breeding sites. We conclude that the microbiota associated with *An. darlingi* immature forms reflect that in their aquatic habitats, but there is also a core mosquito microbiota independent of breeding sites.

## Methods

### *Anopheles darlingi* and breeding water sampling

**Fig. 1:**
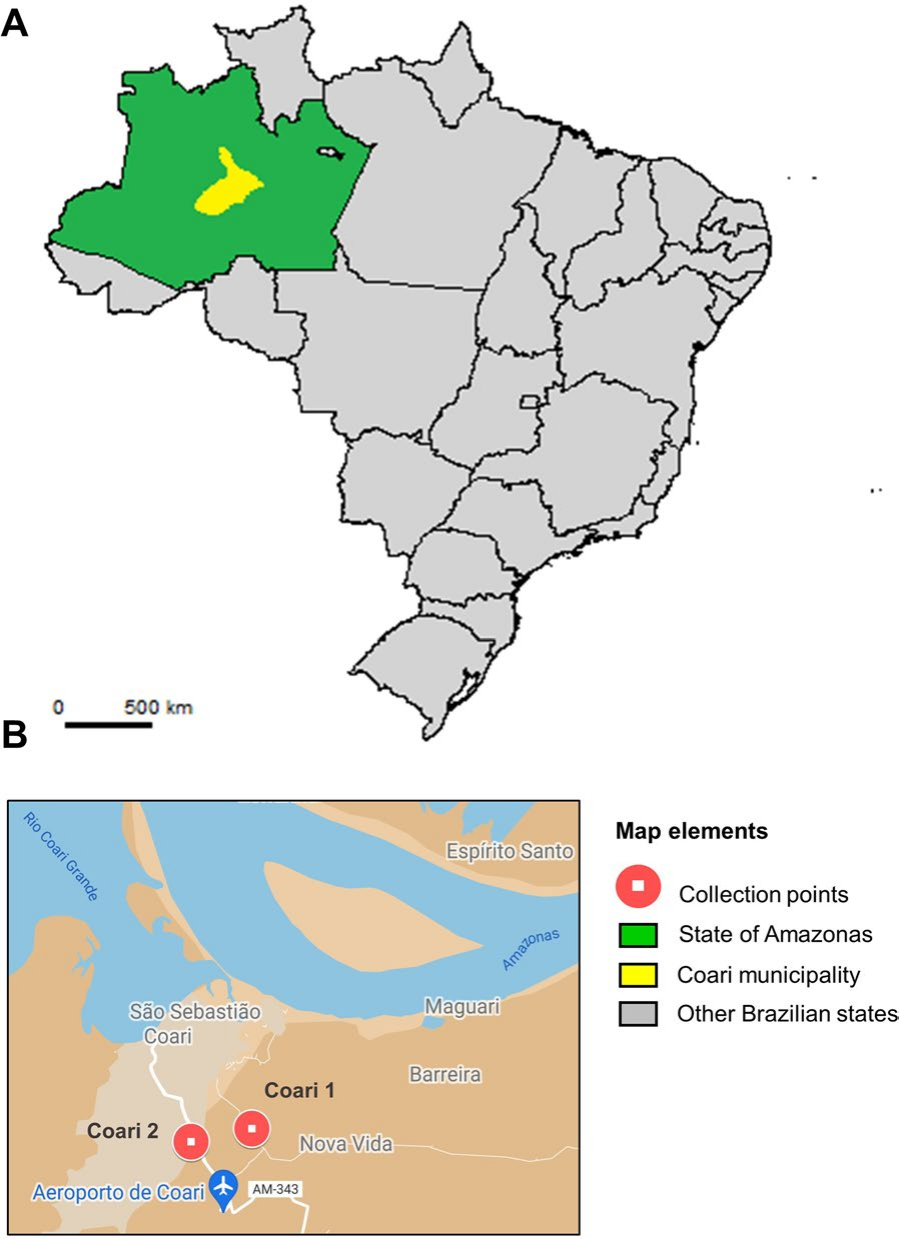
Map showing the geographical location of the collection sites in the Coari municipality, Amazonas state, Brazil. **A** The map of Brazil was taken and modified from gadm.org. **B** The image showing the collection points in Coari was extracted from Google Maps

Water and *An. darlingi* larvae and pupae were collected in artificial ponds and dams used for fish farming in the Coari municipality (Table 1, Fig. 1), an area of active malaria transmission. The two collection sites (Coari 1 and Coari 2) are permanent anopheline mosquito breeding sites located 1.61 km from each other. At both collection sites, samples were obtained from four equidistant sub-sites, approximately 5 m from each other, on the lake/fish farm perimeter.

**Table 1:** Geographical location and number of samples collected at each site in the Coari municipality, Brazilian Amazon

Collection site	Coordinates		Number of samples collected			
	Latitude	Longitude	Larvae 3	Larvae 4	Pupae	Water
Coari 1	S 04° 06. 750′	W 063° 07. 720′	4	3	1	4
Coari 2	S 04° 06. 929′	W 063° 08. 573′	3	4	4	4

*Anopheles darlingi* immature forms were collected at 8:00 for 20 min using a hand dipper and transferred to plastic trays. Larvae and pupae were then picked from the trays with Pasteur pipettes, transferred to 50 ml conical tubes containing breeding water, and stored on ice for transport to the Malaria and Dengue Laboratory (Instituto Nacional de Pesquisas da Amazônia-INPA). Mosquito species were identified immediately after collection based on their morphology using identification keys [39–41]. Larvae and pupae (Adar samples) were rinsed serially for 3 min in the following solutions: sodium hypochlorite (1%), ethanol (70%), and sterile water. Immediately after surface sterilization, the mosquitoes were subjected to DNA extraction.

Surface water samples (900 ml) were collected, along with larvae and pupae, using the same technique and stored on ice in sterile flasks for transport. In the laboratory, each water sample was filtered through Whatman grade 4 filter paper and Millipore membranes of 0.45 μm and 0.22 μm. The retained material was eluted from each filter in 2 ml of autoclaved distilled water and centrifuged for 12 min at 10,000×*g*. The supernatant was discarded and the pellet was submitted for DNA extraction. Altogether, 12 water-derived samples were DNA-extracted per site (Coari 1 and Coari 2), originating from three filters from each of the four sub-sites. DNA sequences of the three filters from each sub-site were pooled together for data processing (Table 1).

### DNA extraction and *16S rRNA* amplification

DNA extraction from water, larvae, and pupae was performed using the innuPREP Plant DNA extraction kit (Analytik Jena) following the manufacturer’s protocol. The recovered DNA was dissolved in 20 μl of nuclease-free water, and the bacterial *16S rRNA* gene was amplified by polymerase chain reaction (PCR) using the primers 27F (5′-AGAGTTTGATCMTGGCTCAG-3′) and 1100R (5′-AGGGTTGCGCTCGTT-3′). The PCR program consisted of an initial denaturation at 95 °C for 5 min, 30 cycles of 94 °C for 1 min, 56 °C for 1 min, and 72 °C for 2 min, followed by a final extension at 72 °C for 10 min. Amplicon production and size were verified by electrophoresis in 1% agarose gel. PCR-negative controls (no template) resulted in no amplification.

### V3–V4 region amplification, barcoding, and sequencing

The PCR products obtained above (~ 1073 bp) were subjected to a two-step PCR method targeting the V3–V4 hypervariable region using the primers 341F (5′-CCTACGGGNGGCWGCAG-3′) and 805R (5′-GACTACHVGGGTATCTAATCC-3′) (for further details see Nilsson and collaborators [31]). In brief, DNA samples were individually PCR-amplified by initial denaturation at 95 °C for 5 min, followed by 20 cycles of 95 °C for 40 s, 53 °C for 40 s, and 72 °C for 1 min, and a final extension at 72 °C for 7 min. The PCR products were diluted in nuclease-free water to a concentration of 0.1–1 ng/μl. In a second PCR, one out of 50 flanking barcode sequence pairs was added to each sample using the same conditions as above, but only for 10 additional cycles. The PCR products were pooled, purified, and eluted in 50 μl nuclease-free water. Finally, the pools were sent to the SNP&SEQ Technology Platform in Uppsala, Sweden (www.sequencing.se) for sequencing. Sequencing libraries were prepared from ~ 10 ng of DNA using the ThruPLEX-FD Prep Kit (R40048-08, Rubicon Genomics) according to the manufacturer’s instructions. The libraries were purified using AMPure XP beads, and the quality was evaluated using the 2200 TapeStation system (Agilent Technologies) and the D1000 ScreenTape assay. The adapter-ligated fragments were quantified by quantitative PCR (qPCR) using the Library quantification kit for Illumina (KAPA Biosystems) on a StepOnePlus instrument (Applied Biosystems/Life Technologies) before cluster generation and sequencing. The pooled DNA samples were paired-end sequenced with 300-base-pair (bp) read length on the MiSeq system (Illumina) using the v3 chemistry according to the manufacturer’s protocols.

### Sequence data processing and generation of OTU table

Paired-end reads were assembled and demultiplexed using Mothur (version 1.36.1), keeping sequences with a difference of fewer than two bases between the primer portion of the read and the primer ([31] and references therein). Further analyses were performed by USEARCH (version 8.1.1861). Reads were filtered to remove low-quality reads using a maximum expected error threshold of 1. The remaining sequences were dereplicated using full-length matching. Clustering of operational taxonomic units (OTUs) was performed using UPARSE with a minimum identity of 97% and discarding singletons and chimeras. To construct the OTU table, the reads before quality filtering and removal of singletons were mapped to the OTUs using a minimum identity of 0.97 to the representative sequence. The taxonomic classification was performed using the UTAX RDP trainset 15 and a pre-trained taxonomy confidence file for a sequence length of 500. Taxonomic annotation was made with a confidence threshold of 0.9. Reads from the three different filters belonging to the same sub-site were added together and treated as one water sample in downstream analyses, yielding eight samples (four sub-sites for each of the two collection sites). OTUs classified as chloroplasts, as well as those that made up < 0.005% of the sequence libraries, were filtered out from further analysis. Moreover, OTUs detected in the negative control sample whose relative abundance was not at least 10 times greater than that observed in the negative control were also removed from the dataset [42].

### Data analysis

All analyses were performed in R software 3.8.2. To visualize the bacterial composition among the mosquito and water samples and their collection sites, bar charts showing the distribution of bacterial phyla, classes, and families were created using the “ggplot2” package [43]. A four-way Venn diagram was generated using the “VennDiagram” package [44]. The observed species richness (S obs) and Shannon diversity index (H) were used to assess alpha diversity using the “phyloseq” package [45]. For this, the OTU table was first rarefied to 1100 reads per sample using the “vegan” package [46]. Alpha diversity metrics were compared between subgroups with a one-way analysis of variance (ANOVA; followed by Tukey’s post hoc test) or Kruskal–Wallis test (followed by Dunn’s post hoc test), depending on normal distribution verification with the Shapiro–Wilk test. Alpha diversity plots and statistical analysis were performed in GraphPad Prism 8.

A Bray–Curtis distance matrix was used for beta diversity analysis using the “vegan” package. Nonmetric multidimensional scaling (NMDS) was performed to visualize the overall dissimilarity in the microbial community structure between the groups, i.e., Adar C1, Adar C2, Water C1, and Water C2. Moreover, a permutational multivariate analysis of variance (PERMANOVA) and an analysis of similarity (ANOSIM) were conducted to explore the significance of the sample type, i.e., mosquitoes (Adar larvae and pupae) and breeding water, and collection site, i.e., Coari 1 and Coari 2, with respect to the bacterial profiles associated with the samples. Indicator species analysis was carried out using the “indicspecies” package [47].

## Results

### Data summary

We collected 19 immature forms of *An. darlingi* and eight water samples from two different collection sites (Table 1) and described their associated microbiota using *16S rRNA* amplicon sequencing. After bioinformatics processing and taxonomic assignment, a total of 226,916 reads were classified into 118 OTUs. The analysis of the negative control showed the presence of bacterial sequences possibly derived from contamination while the samples were being processed. Therefore, OTUs with tenfold higher relative abundance in the negative control than in all *An. darlingi* and water samples combined were filtered out from the dataset. Thus, 114 OTUs exceeding 0.05% in abundance were considered for further analysis. Rarefaction to an even sequencing depth of 1100 reads per sample was used to normalize the dataset. The rarefied OTU table was used to assess alpha diversity metrics.

### Bacterial community profiles

**Fig. 2:**
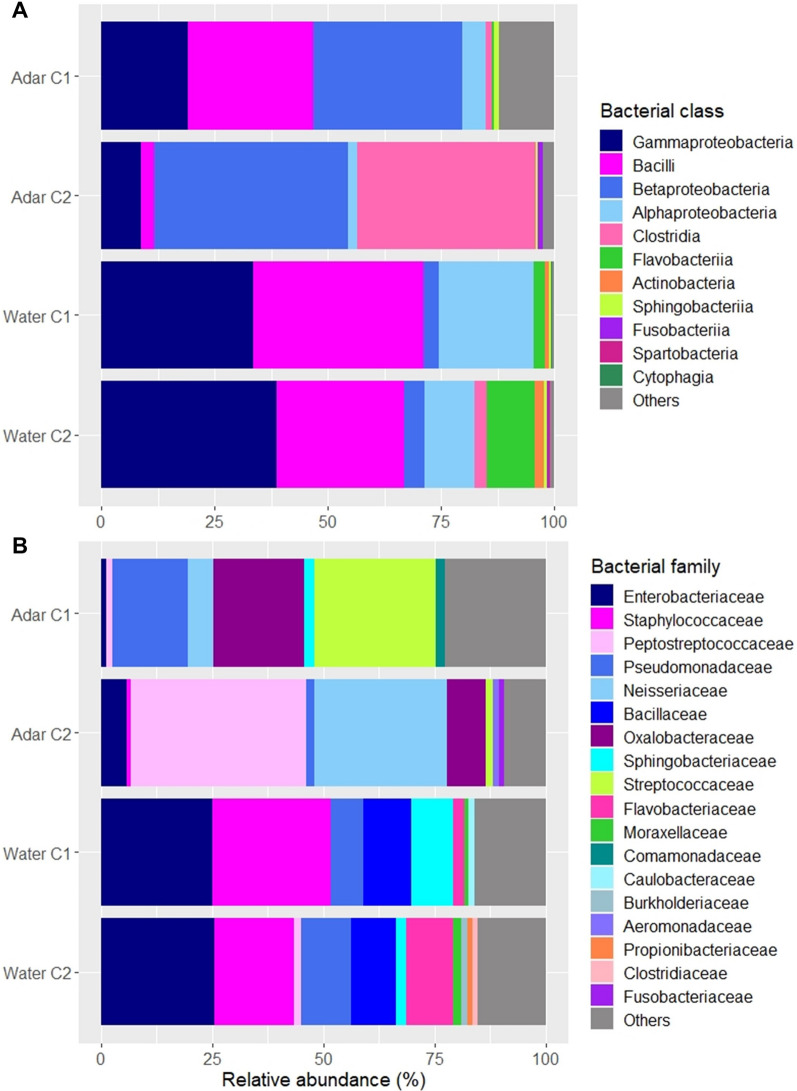
Bacterial community composition of *An. darlingi* larvae and pupae (Adar) and their breeding sites, Coari 1 (C1) and Coari 2 (C2), at **A** class level and **B** family level. Only classes and families making up > 0.1% and > 1%, respectively, in any group of samples, are included. Other classes/families present are clustered as “Others” together with unknown classes/families

Six bacterial phyla and one candidate phylum (Candidatus Saccharibacteria) were identified from *An. darlingi* and water collected from their natural breeding sites (Additional file 1: Fig. S1). Proteobacteria, Firmicutes, Bacteroidetes, Actinobacteria, and Verrucomicrobia were found across all sample groups (Adar C1, Adar C2, Water C1, and Water C2). The most abundant phylum, by average abundance, identified in both Adar and breeding water samples was Proteobacteria (55.91%), followed by Firmicutes (35.92%). Other bacteria phyla made up 7.09% of the dataset, and the remaining 1.05% were unclassified sequences. The candidate phylum Candidatus Saccharibacteria was associated only with breeding water samples. Furthermore, a total of 13 bacterial classes split into 30 bacterial families were observed in the dataset. The five most abundant classes across the Adar samples were Betaproteobacteria (39.28%), Clostridia (26.17%), Gammaproteobacteria (12.33%), Bacilli (11.57%), and Alphaproteobacteria (3.18%), while across the water samples they were Gammaproteobacteria (35.62%), Bacilli (33.72%), Alphaproteobacteria (16.97%), Flavobacteriia (5.77%), and Betaproteobacteria (3.84%) (Fig. 2a). At the family level, *Peptostreptococcaceae*, *Neisseriaceae*, *Oxalobacteraceae*, and *Streptococcaceae* were the most commonly identified taxa in *An. darlingi*, making up 26.14%, 21.47%, 12.82%, and 10.45%, respectively. While the first three families were abundant in the majority of individual mosquitoes, *Streptococcaceae* is prominent in Fig. 2b due to very high abundance in one specimen of the Adar samples and, although detected in most larvae and pupae (data not shown), did not account for more than 1% of the total reads in more than four samples (Additional file 2: Fig. S3). Bacteria within the families *Enterobacteriaceae* (25.15%), *Staphylococcaceae* (23.16%), *Bacillaceae* (10.52%), and *Pseudomonadaceae* (8.85%) were highly abundant across the water collected in breeding sites (Fig. 2b). The bacterial profiles at the class and family levels associated with each individual mosquito and water sample are shown in the Additional file 2: Fig. S2, S3.

The four-way Venn diagram plotting OTU overlap between mosquito and water samples revealed that most of the OTUs identified in each geographical location were shared between *An. darlingi* and their breeding water: 61 and 63 OTUs were shared among samples collected at Coari 1 and Coari 2, respectively (Fig. 4a). Additionally, there were 11 OTUs present in the *An. darlingi* samples but not in the breeding water and 10 OTUs present in the water samples but not in the immature mosquitoes. A table showing the OTUs belonging to each sample type and collection site is presented in Additional file 3: Table S1.

### Alpha diversity of bacterial OTUs

**Fig. 3:**
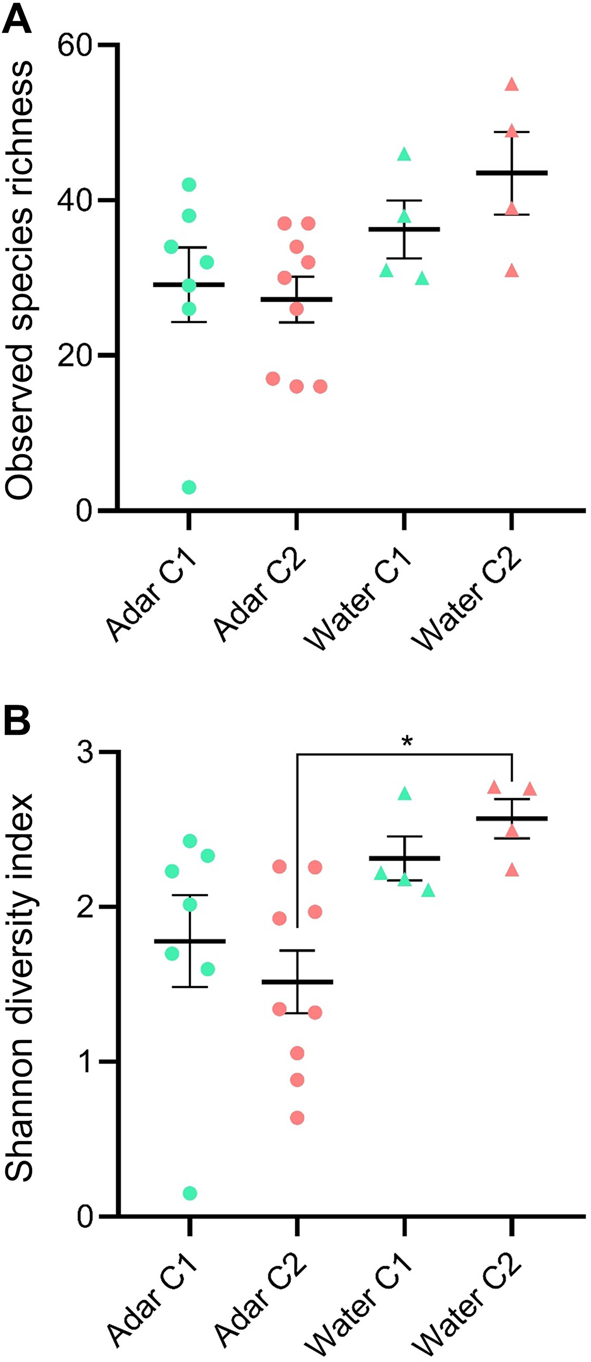
Mean values of alpha diversity metrics. **A** Observed species richness and **B** Shannon diversity index, calculated for the bacterial communities associated with *An. darlingi* larvae and pupae (Adar) and their breeding sites, Coari 1 (C1) and Coari 2 (C2). Error bars represent the standard error of the mean (* *P* < 0.05)

The S obs and H were compared between *An. darlingi* and breeding water sampled at the two different collection sites (Fig. 3). The S obs was used to estimate the number of unique OTUs (richness) present within each sample, while H was used to estimate both OTU richness and evenness (diversity). Overall, we observed higher richness and diversity in water samples than in *An. darlingi*. However, there were no significant differences between subgroups for S obs (ANOVA, *F* (_3, 20_) = 2.745, *P* = 0.07) (Fig. 3a). On the other hand, there were significant differences between subgroups for H (Kruskal–Wallis, *H* = 9.7996, degrees of freedom (*df*) = 3, *P* = 0.0203) (Fig. 3b). The post hoc Dunn test identified statistically significant differences in H between mosquito and water collected at Coari 2 (*P* = 0.0158) (Additional file 4: Table S2). Water samples collected at Coari 2 had the highest OTU richness and diversity (mean S obs = 43.50, mean H = 2.57), while mosquitoes collected at Coari 2 presented the lowest OTU richness and diversity (mean S obs = 27.22, mean H = 1.516).

### Beta diversity

**Fig. 4:**
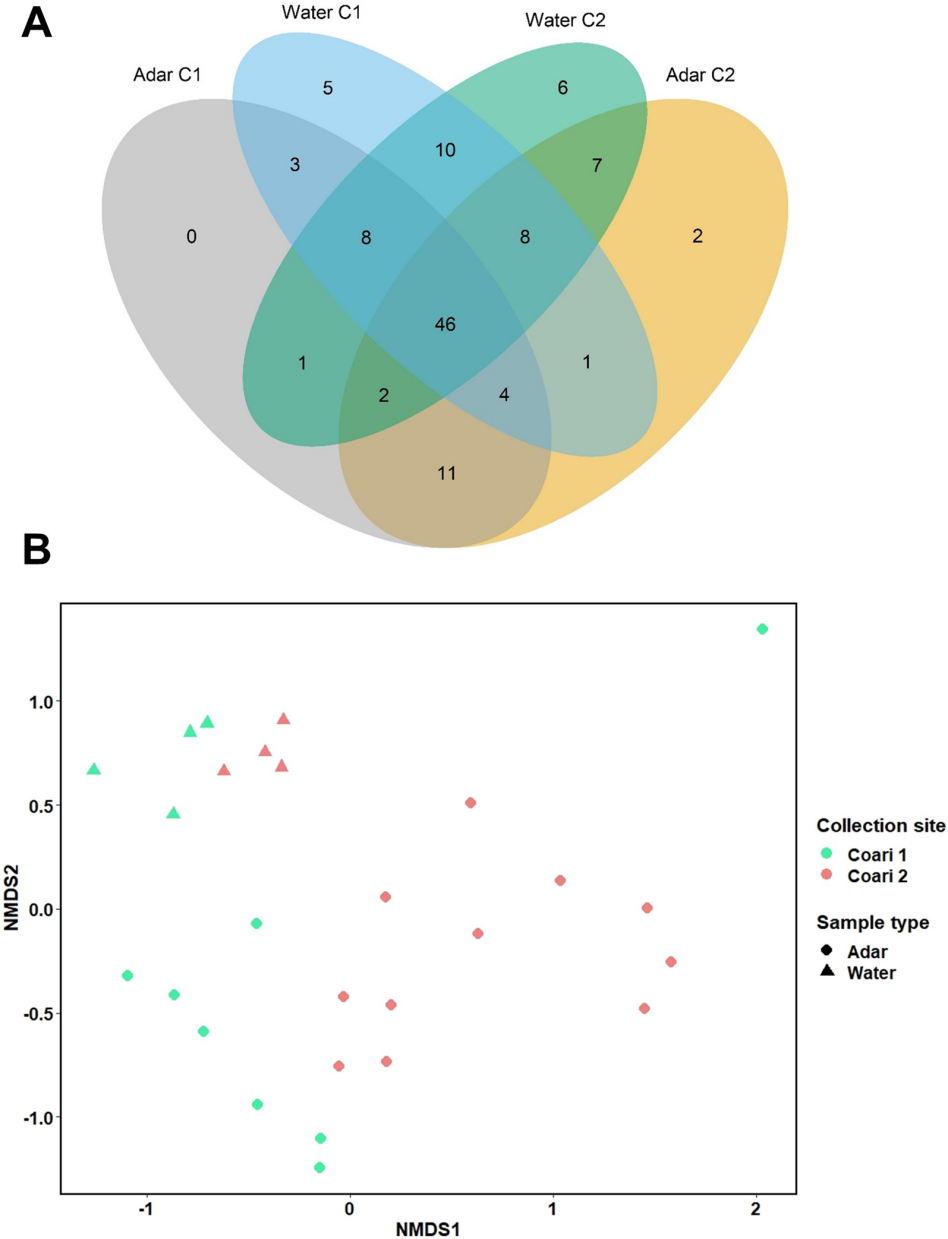
**A** Four-way Venn diagram depicts the number of OTUs that overlap and do not overlap between *An. darlingi* larvae and pupae (Adar) and their breeding water collected at two different geographical locations, Coari 1 (C1) and Coari 2 (C2). **B** Nonmetric multidimensional scaling (NMDS) based on Bray–Curtis distances. Different clustering patterns for each sample type (Adar and water) and collection site (Coari and Coari 2) are represented by a shape code and a color code, respectively

To determine whether the composition and structure of bacterial communities differed between samples, a Bray–Curtis dissimilarity matrix was generated and visualized using an NMDS plot (Fig. 4b). We found that the samples showed a clustering pattern according to their type (Adar and water) and their collection point (Coari 1 and Coari 2). These observations were supported by a PERMANOVA analysis since significant differences were detected in the microbial profiles of the samples according to both their type (*R*^2^ = 0.264, *P* = 0.001) and their collection site (*R*^2^ = 0.08, *P* = 0.014). Furthermore, the ANOSIM test confirmed that there were statistical differences between the bacterial communities of the mosquito and water samples (*R* = 0.4218, *P* = 0.001), as well as the places where they were collected (*R* = 0.2036, *P* = 0.006). A comparison of the microbial profiles associated with mosquitoes belonging to different developmental stages, i.e., larvae and pupae, did not display significant differences (PERMANOVA, *R*^2^ = 0.03047, *P* = 0.310) (Additional file 5: Fig. S4).

### Indicator species analysis

Considering that the samples presented distinct bacterial profiles according to the breeding sites where they were collected, an indicator species analysis was performed to identify which OTUs were driving these differences (Table 2). The majority of bacteria having a significant contribution to the variation seen belong to the phylum Proteobacteria. Indicator OTUs associated with Coari 1 were assigned to the bacterial classes Alphaproteobacteria, Betaproteobacteria, and Sphingobacteriia. Indicator OTUs associated with Coari 2 were mostly assigned to the bacterial class Alphaproteobacteria, followed by Gammaproteobacteria, Betaproteobacteria, Bacilli, Clostridia, and Fusobacteriia.

**Table 2:** OTUs identified as indicator taxa of *An. darlingi* and water samples collected at two different geographical sites

Collection site	OTU	Indval	*P* value	Taxonomy^a^	Top hit^b^
*Coari 1*	OTU22	0.838	0.023	p: Proteobacteria, c: Betaproteobacteria	*Acidovorax*^1^
	OTU9	0.833	0.019	p: Proteobacteria, c: Betaproteobacteria, o: *Burkholderiales*, f: *Oxalobacteraceae*	*Herbaspirillum*^2^
	OTU306	0.816	0.001	p: Proteobacteria, c: Alphaproteobacteria, o: *Rhizobiales*, f: *Rhizobiaceae*, g: *Rhizobium*	
	OTU20	0.804	0.030	p: Proteobacteria, c: Alphaproteobacteria, o: *Sphingomonadales*, f: *Sphingomonadaceae*	*Novosphingobium*^2^
	OTU15	0.800	0.039	p: Proteobacteria, c: Alphaproteobacteria	*Sphingorhabdus*^2^
	OTU36	0.777	0.008	p: Proteobacteria, c: Alphaproteobacteria	*Caulobacter*^1^
	OTU 270	0.726	0.033	p: Proteobacteria, c: Betaproteobacteria	*Undibacterium*^1^
	OTU3	0.707	0.003	p: Proteobacteria, c: Betaproteobacteria, o: *Burkholderiales*, f: *Oxalobacteraceae*	*Undibacterium*^1^
	OTU136	0.702	0.042	p: Bacteroidetes, c: Sphingobacteriia	
*Coari 2*	OTU87	0.948	0.001	p: Firmicutes, c: Clostridia, o: *Clostridiales*, f: *Peptostreptococcaceae*, g:* Clostridium*	
	OTU19	0.931	0.013	p: Proteobacteria, c: Alphaproteobacteria, o: *Rhizobiales*	*Methylocystis*^2^
	OTU98	0.730	0.003	p: Proteobacteria, c: Gammaproteobacteria, o: *Enterobacteriales*, f: *Enterobacteriaceae*, g:* Edwardsiella*	
	OTU57	0.730	0.005	p: Firmicutes, c: Bacilli, o: *Bacillales*, f: *Bacillaceae*, g:* Bacillus*	
	OTU300	0.730	0.006	p: Fusobacteria, c: Fusobacteriia, o: *Fusobacteriales*, f: *Fusobacteriaceae*, g: *Cetobacterium*	
	OTU318	0.719	0.026	p: Proteobacteria, c: Betaproteobacteria, o: *Neisseriales*, f: *Neisseriaceae*, g:* Microvirgula*	
	OTU63	0.700	0.019	p: Proteobacteria, c: Alphaproteobacteria	
	OTU71	0.684	0.035	p: Proteobacteria, c: Gammaproteobacteria, o: *Enterobacteriales*, f: *Enterobacteriaceae*	*Plesiomonas*^2^
	OTU89	0.577	0.049	p: Proteobacteria, c: Alphaproteobacteria, o: *Rhizobiales*	
	OTU115	0.577	0.035	p: Proteobacteria	

*OTU* operational taxonomic unit, *Indval* indicator value

^a^p, c, o, f, and g refer to the taxonomic levels phylum, class, order, family, and genus, respectively

^b^OTUs not identified at the genus level during taxonomic assignment were submitted for BLAST searches against the database of the Integrated Microbial Genomes and Microbiomes (IMG/M).

^1^ Bacterial genus with 97–98% identity

^2^ Bacterial genus with 99–100% identity

## Discussion

The composition and diversity of mosquito microbiota are closely related to the environment with which these insects interact during their different life stages. Some bacteria present in aquatic habitats are able to colonize mosquito larvae after egg-hatching while they are immature forms and recently emerged adults just after completing metamorphosis. Therefore, breeding sites are determinants in the structure of the microbial communities associated with mosquitoes. In this study, we described the bacterial communities of *An. darlingi* larvae and pupae and their rearing water collected at two different sites. *Anopheles darlingi* and water presented distinct microbial profiles, which could be related to a decrease in the richness and diversity of *An. darlingi* microbiota. Furthermore, we showed that the bacterial profiles of our samples could be discriminated according to the geographical location where they were collected.

In Colombia, the three dominant bacterial classes identified in *An. darlingi* larvae, adults, and breeding water sampled in different malaria-endemic regions belong to the classes Actinobacteria, Betaproteobacteria, and Gammaproteobacteria, which vary across sample types [30]. In particular, the most abundant classes in larval samples were Betaproteobacteria and Gammaproteobacteria. This is in line with our observations, as the aforementioned bacteria were highly abundant in our immature mosquitoes, making up 51.6% of the reads. At the family level, *Oxalobacteraceae*, one of the most abundant taxa identified in our Adar samples, has been reported in *An. darlingi* larvae and adults collected in the Peruvian and Brazilian Amazon basins [32, 35]. To our knowledge, bacteria belonging to the families *Peptostreptococcaceae*, *Neisseriaceae*, and *Streptococcaceae* are described for the first time in association with this mosquito species. *Peptostreptococcaceae* and *Streptococcaceae* have been reported in *Anopheles* collected in Vietnam and Thailand [48, 49]. As bacteria naturally acquired by mosquitoes can influence their susceptibility to get infected and transmit pathogens, it is worth mentioning that both bacterial families were identified in one *Anopheles minimus* infected with *P. vivax* [49]. *Enterobacteriaceae*, a less abundant family reported in our samples, has previously been found in *An. darlingi* eggs, larvae, pupae, and adults, including midgut and feces [30, 32, 34, 35]. Furthermore, *Enterobacteriaceae* are predominant in *Anopheles gambiae* and *An. darlingi* mosquitoes infected with malaria parasites [4, 33].

The bacterial communities associated with the breeding sites of *An. darlingi* in Colombia and Brazil are mostly composed of members of the classes Gammaproteobacteria, Bacilli, and Betaproteobacteria [30, 31]. We also found a high abundance of these bacteria in the Coari water samples. Moreover, *Enterobacteriaceae*, *Staphylococcaceae*, and *Pseudomonadaceae*, three of the dominant families described here, appear to be common members of the aquatic habitats of *An. darlingi*, as these bacteria have also been found in breeding water collected in Manaus [31].

Mosquito breeding sites are complex environments with several biotic and abiotic features generating suitable conditions that promote the development of vast communities of microbes [50, 51]. Mosquito larvae are usually non-selective filter feeders of microorganisms and organic particles suspended in water [52]. Consequently, most bacteria present in aquatic habitats could likely pass through larval brushes and enter the gut [30]. Despite this, we observed that early-stage *An. darlingi* contained only a proportion of the OTUs present in the water they were collected from. We also found differences in the abundance of certain bacterial taxa between water and Adar samples. Our observations support the hypothesis that although the microbiota colonizing immature forms is acquired from aquatic niches, the larval gut is a more selective environment [28]. Moreover, we observed a greater richness and diversity in breeding water compared to Adar samples. Our results reinforce previously reported findings suggesting that larvae filter many bacteria and are colonized by a subset of the microorganisms with which they interact and/or on which they feed [5, 7–9, 30, 38]. Which bacterial taxa prevail will depend not only on host control, but also on their ability to compete in the complex midgut microbial community [53]. Bacteria that establish symbiotic associations early during larval development are likely to inhibit colonization by additional taxa [8, 54]. Interestingly, there were OTUs present in the Adar samples but not identified in the aquatic habitat where they developed. It should be considered that these OTUs could also be present in water but at such a low frequency that our sequencing method did not detect them. In addition, the low number of water samples per collection site may not have captured the true taxonomic diversity. Conversely, our findings could indicate that these microbes were transferred vertically from gravid females to their progeny. We observed that OTUs assigned to the families *Streptococcaceae* and *Sphingomonadaceae* were only associated with Adar samples (Additional file 3: Table S1). It has been proposed that mosquito females can add key microbial associates to their breeding sites during egg-laying [7]. In addition, some members of the bacterial community associated with early-stage mosquitoes can be transstadially transmitted to adults [55]. Members of *Streptococcaceae* and *Sphingomonadaceae* have been reported as significantly more abundant in insecticide-resistant *An. gambiae* mosquitoes [56]. Therefore, whether these bacteria can be transferred from aquatic forms to adult *An*. *darlingi* deserves attention.

In terms of beta diversity, we identified significant differences in the microbial profiles and bacterial communities across sample types and collection sites. This is in agreement with our initial hypothesis that the microbiota of early-stage mosquitoes mirror that in the aquatic niches where they develop. Considering this, we investigated which OTUs displayed high specificity and fidelity toward the breeding site they were collected from. Indicator species analysis showed that the class Sphingobacteriia was a bacterial signature of the samples collected at Coari 1. Bacteria belonging to this taxon are one of the most common classes identified across all developmental stages of *Aedes albopictus* [57]. Members of this bacterial class have also been isolated from *An. gambiae* larvae and pupae, and their vertical and horizontal transfer has been reported [55]. Several different genera belonging to the order *Burkholderiales* were specific for Coari 1, while the order *Clostridiales* represented Coari 2. In a study that aimed to identify patterns between nutrient contents and microbial composition in larval habitats and bacterial communities associated with *Culex nigripalpus*, the authors noticed that mosquitoes originating from low-nutrient habitats were associated with *Burkholderiales,* but those from high-nutrient habitats were associated with *Clostridiales* [58]. Whether these observations extend to *An. darlingi* breeding sites could be further investigated, but translated into the current study, would indicate that Coari 2 would be more nutrient-rich than Coari 1. However, a recent study suggests that this neotropical anopheline is opportunistic and can develop in breeding sites harboring different bacteria [31]. Different members of *Rhizobiales* were indicators of Coari 1 and Coari 2, respectively; these bacteria have been identified as intestinal symbionts aiding the acquisition of nitrogen in some herbivorous ants [59]. In particular, *Rhizobium* was listed as part of the core microbiota in different *Anopheles* mosquito tissues [28]. *Bacillus*, as an indicator species of Coari 2, has been isolated from *An. darlingi* immature and adult mosquitoes as well as their breeding sites [35]. Therefore, this genus seems to have established a close association with this mosquito host.

## Conclusions

Our findings show that the microbiota of immature mosquitoes reflects the environment in which they live and that, based on bacterial profiles, mosquitoes can be discriminated into different populations. The study of the bacterial communities associated with *An. darlingi* and their breeding sites may contribute to obtaining a more depurated list of microbial candidates that could be exploited in novel control strategies based on mosquito–microbiota interactions.

## Supplementary Information

**Additional file 1: Figure S1.** Bacterial community composition of *An. darlingi* larvae and pupae (Adar) and their breeding sites, Coari 1 (C1) and Coari 2 (C2), at the phylum level. “Unknown” = unknown phylum.

**Additional file 2: Figure S2.** Bacterial community composition of *An. darlingi* larvae (Adar L) and pupae (Adar P) and their breeding sites, Coari 1 (C1) and Coari 2 (C2), at the class level. Only classes making up > 0.1% are included. Other classes present are clustered as “Others” together with unknown classes. **Figure S3.** Bacterial community composition of *An. darlingi* larvae (Adar L) and pupae (Adar P) and their breeding sites, Coari 1 (C1) and Coari 2 (C2), at the family level. Only families making up > 1% are included. Other families present are clustered as “Others” together with unknown families.

**Additional file 3: Table S1.** Operational taxonomic units with their taxonomic affiliation identified in *An. darlingi* (Adar) and their breeding water collected at two different geographical points, Coari 1 (C1) and Coari 2 (C2).

**Additional file 4: Table S2.** Post hoc Dunn test identified statistically significant differences in the Shannon diversity index between *An. darlingi* and water samples collected at Coari 2 (* *P* < 0.05).

**Additional file 5: Figure S4.** Nonmetric multidimensional scaling (NMDS) based on Bray–Curtis distances. The clustering patterns for each sample type (Adar larva, Adar pupa, and water) and collection site (Coari and Coari 2) are represented by a shape code and a color code, respectively.

## Data Availability

All raw sequence data are available in the European Nucleotide Archive (ENA) at EMBL-EBI under accession number PRJEB56870.

## Abbreviations

*16S rRNA*: 16S ribosomal RNA
Adar: *Anopheles darlingi*
PCR: Polymerase chain reaction
OTU: Operational taxonomic unit
S obs: Observed species richness
H: Shannon diversity index
ANOVA: Analysis of variance
NMDS: Nonmetric multidimensional scaling
PERMANOVA: Permutational multivariate analysis of variance
ANOSIM: Analysis of similarity
