# Supplementary material for: Comparative assessment of the bacterial communities associated with Anopheles darlingi immature stages and their breeding sites in the Brazilian Amazon
Source: Parasit Vectors. 2023 May 1;16:156. doi: 10.1186/s13071-023-05749-6 (PMC10150499; doi:10.1186/s13071-023-05749-6)
Supplement: Supplementary file 1 — Additional file 1: Figure S1. Bacterial community composition of An. darlingi larvae and pupae (Adar) and their breeding sites, Coari 1 (C1) and Coari 2 (C2), at the phylum level. “Unknown” = unknown phylum. [file 13071_2023_5749_MOESM1_ESM.pdf]

Additional file 1

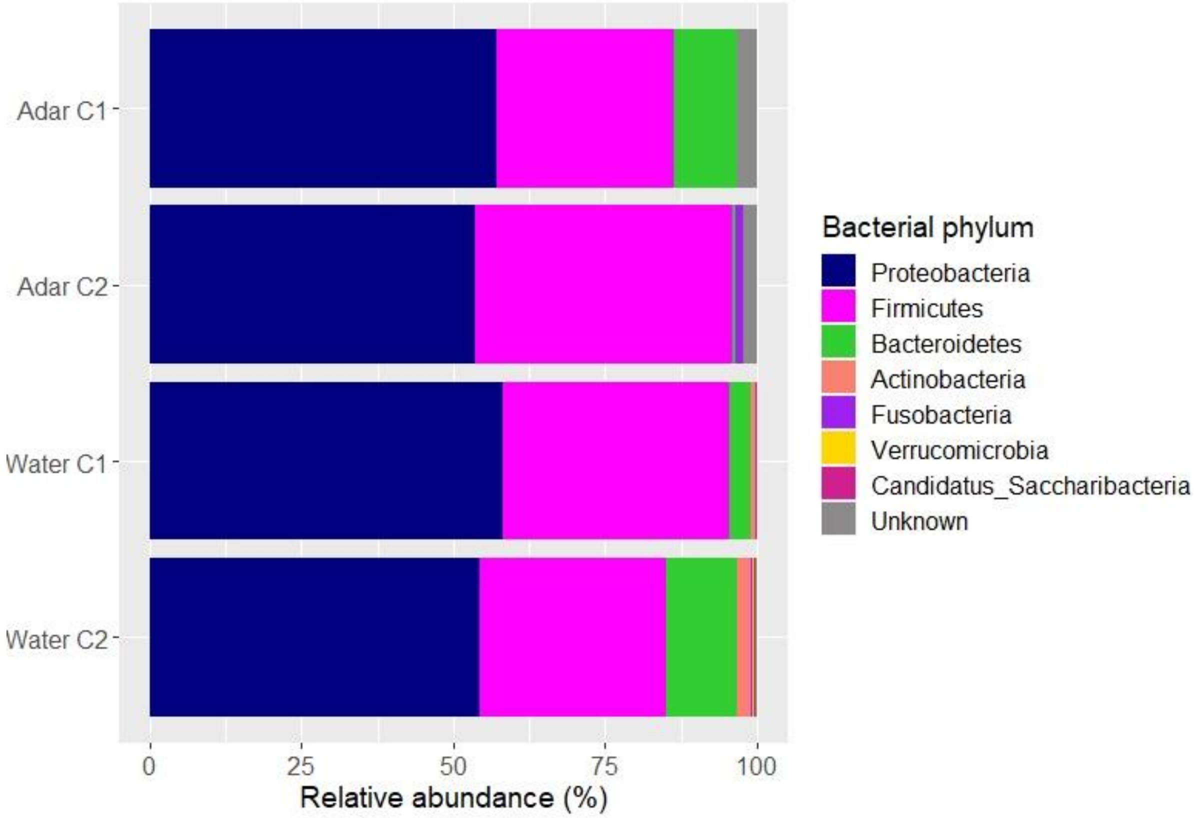

Fig. S1. Bacterial community composition of *An. darlingi*, larvae and pupae (Adar), and their breeding sites, Coari 1 (C1) and Coari 2 (C2), at phylum level. “Unknown” = unknown phylum.
