# Supplementary material for: Comparative assessment of the bacterial communities associated with Anopheles darlingi immature stages and their breeding sites in the Brazilian Amazon
Source: Parasit Vectors. 2023 May 1;16:156. doi: 10.1186/s13071-023-05749-6 (PMC10150499; doi:10.1186/s13071-023-05749-6)
Supplement: Supplementary file 2 — Additional file 2: Figure S2. Bacterial community composition of An. darlingi larvae (Adar L) and pupae (Adar P) and their breeding sites, Coari 1 (C1) and Coari 2 (C2), at the class level. Only classes making up > 0.1% are included. Other classes present are clustered as “Others” together with unknown classes. Figure S3. Bacterial community composition of An. darlingi larvae (Adar L) and pupae (Adar P) and their breeding sites, Coari 1 (C1) and Coari 2 (C2), at the family level. Only families making up > 1% are included. Other families present are clustered as “Others” together with unknown families. [file 13071_2023_5749_MOESM2_ESM.pdf]

Additional file 2

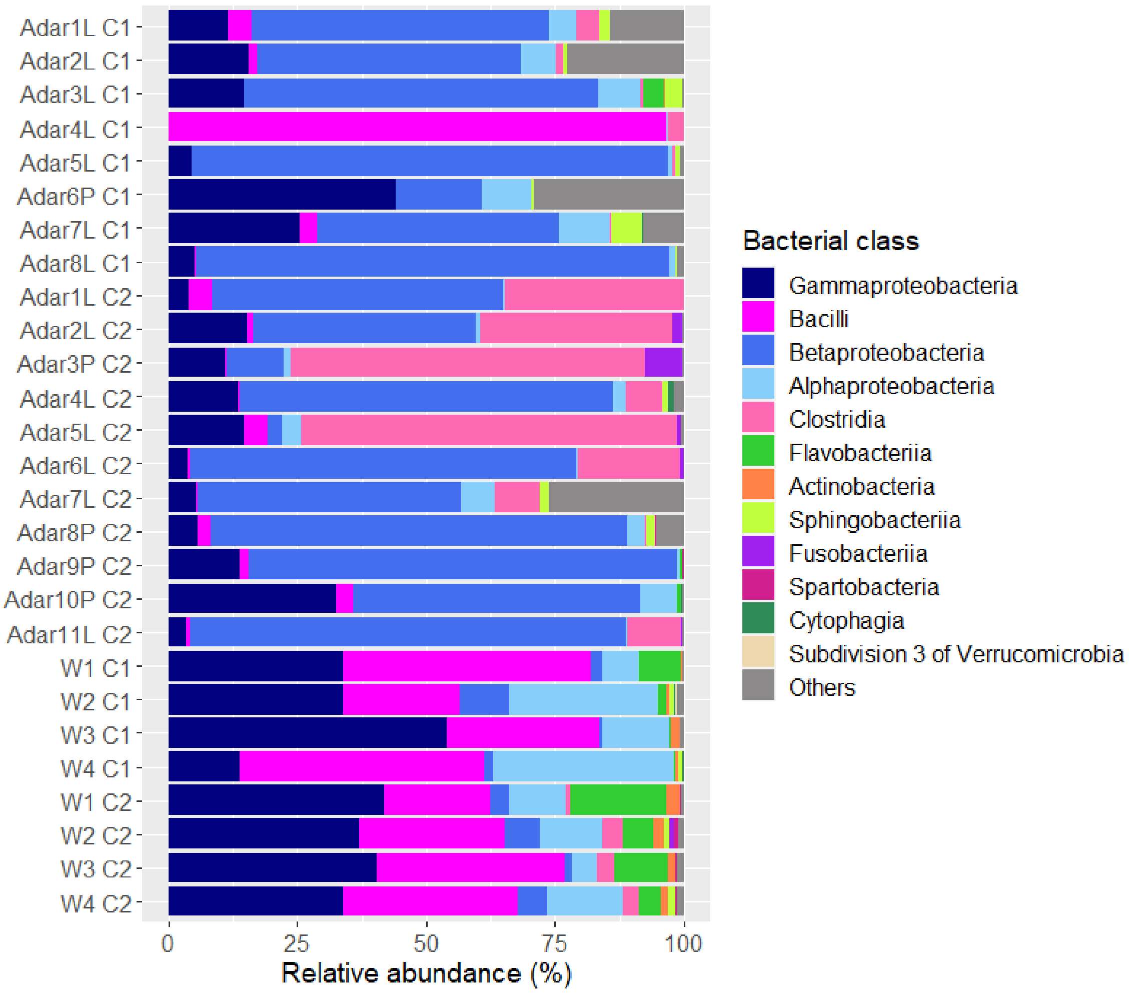

Fig. S2. Bacterial community composition of *An. darlingi*, larvae (Adar L) and pupae (Adar P), and their breeding sites, Coari 1 (C1) and Coari 2 (C2), at class level. Only classes making up > 0.1% are included. Other classes present are clustered as “Others” together with unknown classes.

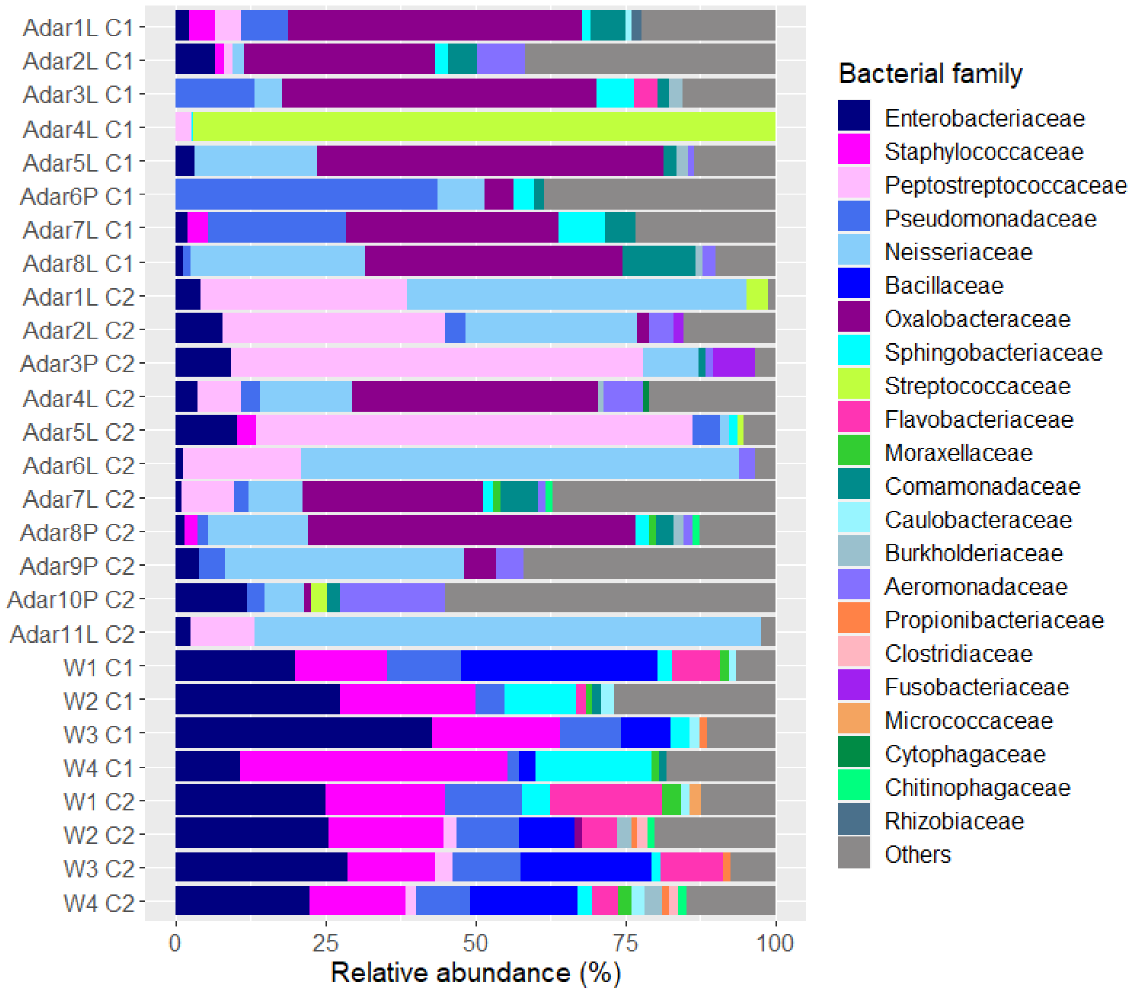

Fig. S3. Bacterial community composition of *An. darlingi*, larvae (Adar L) and pupae (Adar P), and their breeding sites, Coari 1 (C1) and Coari 2 (C2), at family level. Only families making up > 1% are included. Other families present are clustered as “Others” together with unknown families.
