# Supplementary material for: Comparative assessment of the bacterial communities associated with Anopheles darlingi immature stages and their breeding sites in the Brazilian Amazon
Source: Parasit Vectors. 2023 May 1;16:156. doi: 10.1186/s13071-023-05749-6 (PMC10150499; doi:10.1186/s13071-023-05749-6)
Supplement: Supplementary file 3 — Additional file 3: Table S1. Operational taxonomic units with their taxonomic affiliation identified in An. darlingi (Adar) and their breeding water collected at two different geographical points, Coari 1 (C1) and Coari 2 (C2). [file 13071_2023_5749_MOESM3_ESM.pdf]

Additional file 3

Table S1. Operational taxonomic units with their taxonomic affiliation identified in *An. darlingi* (Adar) and their breeding water collected at two different geographical points, Coari 1 (C1) and Coari 2 (C2).

OTU	Phylum	Class	Family	Adar C1	Water C1	Adar C2	Water C2
OTU283	Proteobacteria	Gammaproteobacteria	Enterobacteriaceae	Y	Y	Y	Y
OTU87	Firmicutes	Clostridia	Peptostreptococcaceae	Y	Y	Y	Y
OTU272	Firmicutes	Bacilli	Staphylococcaceae	Y	Y	Y	Y
OTU40	Proteobacteria	Gammaproteobacteria	Pseudomonadaceae	Y	Y	Y	Y
OTU318	Proteobacteria	Betaproteobacteria	Neisseriaceae	Y	N	Y	Y
OTU23	Firmicutes	Bacilli	Bacillaceae	N	Y	Y	Y
OTU224	Firmicutes	Bacilli	Staphylococcaceae	Y	Y	Y	Y
OTU75	Proteobacteria	Betaproteobacteria	Oxalobacteraceae	Y	Y	Y	Y
OTU121	Firmicutes	Bacilli	Streptococcaceae	Y	N	Y	N
OTU240	Bacteroidetes	Flavobacteriia	Flavobacteriaceae	N	Y	Y	Y
OTU5	Proteobacteria	Gammaproteobacteria	Enterobacteriaceae	Y	Y	Y	Y
OTU30	Proteobacteria	Alphaproteobacteria	Sphingomonadaceae	Y	Y	Y	Y
OTU129	Proteobacteria	Alphaproteobacteria		Y	Y	Y	Y
OTU20	Proteobacteria	Alphaproteobacteria	Sphingomonadaceae	Y	Y	Y	Y
OTU48	Bacteroidetes			Y	Y	Y	Y
OTU15	Proteobacteria	Alphaproteobacteria		Y	Y	Y	Y
OTU56	Proteobacteria	Betaproteobacteria	Neisseriaceae	Y	Y	Y	N
OTU269	Proteobacteria	Betaproteobacteria		Y	Y	Y	Y
OTU188				Y	N	Y	N
OTU59	Proteobacteria	Gammaproteobacteria	Moraxellaceae	Y	Y	Y	Y

OTU1	Proteobacteria	Betaproteobacteria	Comamonadaceae	Y	Y	Y	Y
OTU84	Proteobacteria	Alphaproteobacteria		N	Y	N	Y
OTU13	Proteobacteria	Gammaproteobacteria	Aeromonadaceae	Y	Y	Y	Y
OTU26	Proteobacteria	Alphaproteobacteria		Y	Y	N	Y
OTU17	Proteobacteria	Betaproteobacteria	Burkholderiaceae	Y	Y	Y	Y
OTU19	Proteobacteria	Alphaproteobacteria		Y	Y	Y	Y
OTU44	Actinobacteria	Actinobacteria	Propionibacteriaceae	Y	Y	Y	Y
OTU3	Proteobacteria	Betaproteobacteria	Oxalobacteraceae	Y	Y	N	N
OTU64	Proteobacteria	Alphaproteobacteria		Y	Y	Y	Y
OTU296	Proteobacteria	Alphaproteobacteria	Caulobacteraceae	Y	Y	Y	Y
OTU315	Firmicutes	Clostridia	Clostridiaceae	Y	Y	Y	Y
OTU300	Fusobacteria	Fusobacteriia	Fusobacteriaceae	N	N	Y	Y
OTU157	Proteobacteria	Betaproteobacteria		Y	Y	N	Y
OTU160	Proteobacteria	Betaproteobacteria		Y	Y	Y	Y
OTU69	Proteobacteria	Alphaproteobacteria	Sphingomonadaceae	Y	Y	Y	Y
OTU189	Proteobacteria	Betaproteobacteria		Y	N	Y	N
OTU45	Proteobacteria	Alphaproteobacteria		Y	Y	Y	Y
OTU323	Proteobacteria	Betaproteobacteria		Y	Y	Y	Y
OTU105	Proteobacteria	Gammaproteobacteria	Pseudomonadaceae	Y	Y	N	Y
OTU35	Actinobacteria	Actinobacteria	Micrococcaceae	Y	Y	N	Y
OTU12	Proteobacteria	Gammaproteobacteria	Enterobacteriaceae	Y	Y	Y	Y
OTU22	Proteobacteria	Betaproteobacteria		Y	Y	Y	Y
OTU212	Proteobacteria	Alphaproteobacteria	Caulobacteraceae	N	Y	N	Y
OTU41	Bacteroidetes	Sphingobacteriia	Chitinophagaceae	Y	Y	Y	Y
OTU104	Proteobacteria	Alphaproteobacteria	Caulobacteraceae	Y	Y	Y	Y
OTU98	Proteobacteria	Gammaproteobacteria	Enterobacteriaceae	N	N	Y	Y
OTU16	Verrucomicrobia	Spartobacteria		Y	Y	Y	Y
OTU82				Y	Y	Y	N
OTU255	Proteobacteria	Gammaproteobacteria	Enterobacteriaceae	Y	Y	Y	Y

OTU33	Proteobacteria	Gammaproteobacteria	Moraxellaceae	Y	Y	Y	Y
OTU294	Proteobacteria	Alphaproteobacteria		N	Y	Y	Y
OTU81	Bacteroidetes	Sphingobacteriia	Chitinophagaceae	Y	Y	Y	Y
OTU91	Proteobacteria	Betaproteobacteria		N	Y	Y	Y
OTU10	Proteobacteria	Alphaproteobacteria	Methylobacteriaceae	N	Y	Y	Y
OTU9	Proteobacteria	Betaproteobacteria	Oxalobacteraceae	Y	Y	Y	Y
OTU178	Bacteroidetes	Sphingobacteriia	Sphingobacteriaceae	Y	N	Y	N
OTU155	Firmicutes	Bacilli	Enterococcaceae	N	Y	Y	N
OTU63	Proteobacteria	Alphaproteobacteria		N	Y	Y	Y
OTU83	Actinobacteria	Actinobacteria	Nocardiaceae	N	Y	N	Y
OTU136	Bacteroidetes	Sphingobacteriia		Y	Y	Y	Y
OTU169	Bacteroidetes	Cytophagia	Cytophagaceae	Y	Y	Y	Y
OTU46	Proteobacteria	Betaproteobacteria		Y	Y	Y	Y
OTU54	Proteobacteria	Betaproteobacteria	Comamonadaceae	Y	Y	Y	Y
OTU71	Proteobacteria	Gammaproteobacteria	Enterobacteriaceae	Y	N	Y	N
OTU42	Bacteroidetes	Sphingobacteriia		Y	Y	Y	Y
OTU96	Bacteroidetes	Flavobacteriia	Flavobacteriaceae	N	Y	Y	Y
OTU36	Proteobacteria	Alphaproteobacteria		Y	Y	N	Y
OTU60	Bacteroidetes	Flavobacteriia	Flavobacteriaceae	Y	N	N	Y
OTU270	Proteobacteria	Betaproteobacteria		Y	Y	Y	N
OTU306	Proteobacteria	Alphaproteobacteria	Rhizobiaceae	Y	Y	N	N
OTU184	Bacteroidetes	Sphingobacteriia		Y	Y	N	Y
OTU137	Proteobacteria	Betaproteobacteria	Burkholderiaceae	Y	Y	N	Y
OTU51	Actinobacteria			Y	Y	Y	Y
OTU247	Bacteroidetes			N	N	N	Y
OTU290	Proteobacteria			N	Y	N	Y
OTU67	Candidatus Saccharibacteria			N	Y	N	Y
OTU94	Proteobacteria	Betaproteobacteria		Y	N	Y	N

OTU220	Proteobacteria	Gammaproteobacteria		N	Y	N	Y
OTU102	Proteobacteria	Alphaproteobacteria		Y	Y	N	Y
OTU25				N	Y	N	Y
OTU297				Y	Y	N	N
OTU161				Y	Y	Y	N
OTU312	Actinobacteria	Actinobacteria		N	Y	N	N
OTU57	Firmicutes	Bacilli	Bacillaceae	N	N	Y	Y
OTU172	Verrucomicrobia	Subdivision3		N	Y	N	N
OTU185	Firmicutes	Bacilli	Bacillaceae	N	Y	N	Y
OTU85	Proteobacteria	Alphaproteobacteria	Methylobacteriaceae	Y	Y	Y	Y
OTU273	Actinobacteria	Actinobacteria	Microbacteriaceae	Y	Y	Y	Y
OTU86	Proteobacteria	Alphaproteobacteria		Y	Y	Y	Y
OTU68	Bacteroidetes	Sphingobacteriia		Y	Y	Y	Y
OTU222				N	Y	N	Y
OTU89	Proteobacteria	Alphaproteobacteria		N	N	Y	Y
OTU275	Fusobacteria	Fusobacteriia	Leptotrichiaceae	N	N	N	Y
OTU165	Proteobacteria	Alphaproteobacteria		Y	N	Y	Y
OTU115	Proteobacteria			N	N	Y	N
OTU311	Verrucomicrobia	Spartobacteria		N	N	N	Y
OTU197	Firmicutes	Bacilli	Leuconostocaceae	N	Y	N	N
OTU38	Actinobacteria			N	Y	N	N
OTU124				N	N	Y	N
OTU234	Proteobacteria	Gammaproteobacteria	Pseudomonadaceae	N	Y	N	N
OTU282	Bacteroidetes	Sphingobacteriia	Chitinophagaceae	Y	N	Y	N
OTU95	Proteobacteria	Betaproteobacteria	Rhodocyclaceae	Y	N	Y	N
OTU65	Proteobacteria	Betaproteobacteria		Y	N	Y	N
OTU92	Proteobacteria	Betaproteobacteria	Burkholderiaceae	N	Y	N	Y
OTU125	Proteobacteria	Betaproteobacteria		N	N	Y	Y
OTU305	Bacteroidetes	Sphingobacteriia	Sphingobacteriaceae	Y	N	Y	N

OTU316	Proteobacteria			N	Y	Y	Y
OTU253	Firmicutes	Clostridia	Clostridiales	N	N	N	Y
OTU190	Proteobacteria			Y	Y	Y	Y
OTU53	Firmicutes	Bacilli	Carnobacteriaceae	N	N	N	Y
OTU226	Proteobacteria	Gammaproteobacteria	Enterobacteriaceae	N	N	Y	Y
OTU78	Proteobacteria	Betaproteobacteria	Neisseriaceae	N	N	Y	Y
OTU245	Actinobacteria	Actinobacteria		N	N	N	Y
OTU313	Bacteroidetes	Sphingobacteriia	Sphingobacteriaceae	Y	N	Y	N
