# Supplementary material for: Comparative assessment of the bacterial communities associated with Anopheles darlingi immature stages and their breeding sites in the Brazilian Amazon
Source: Parasit Vectors. 2023 May 1;16:156. doi: 10.1186/s13071-023-05749-6 (PMC10150499; doi:10.1186/s13071-023-05749-6)
Supplement: Supplementary file 4 — Additional file 4: Table S2. Post hoc Dunn test identified statistically significant differences in the Shannon diversity index between An. darlingi and water samples collected at Coari 2 (* P < 0.05). [file 13071_2023_5749_MOESM4_ESM.pdf]

Additional file 4

Table S2. Post hoc Dunn test identified statistically significant differences in Shannon diversity index between *An. darlingi* (Adar) and water samples collected at Coari 2 (* = $P < 0.05$).

Dunn's multiple comparisons test	Adjusted P Value
Adar C1 vs. Adar C2	>0.9999
Adar C1 vs. Water C1	>0.9999
Adar C1 vs. Water C2	0.2003
Adar C2 vs. Water C1	0.5889
Adar C2 vs. Water C2	0.0158 *
Water C1 vs. Water C2	>0.9999
