# Supplementary material for: Comparative assessment of the bacterial communities associated with Anopheles darlingi immature stages and their breeding sites in the Brazilian Amazon
Source: Parasit Vectors. 2023 May 1;16:156. doi: 10.1186/s13071-023-05749-6 (PMC10150499; doi:10.1186/s13071-023-05749-6)
Supplement: Supplementary file 5 — Additional file 5: Figure S4. Nonmetric multidimensional scaling (NMDS) based on Bray–Curtis distances. The clustering patterns for each sample type (Adar larva, Adar pupa, and water) and collection site (Coari and Coari 2) are represented by a shape code and a color code, respectively. [file 13071_2023_5749_MOESM5_ESM.pdf]

Additional file 5

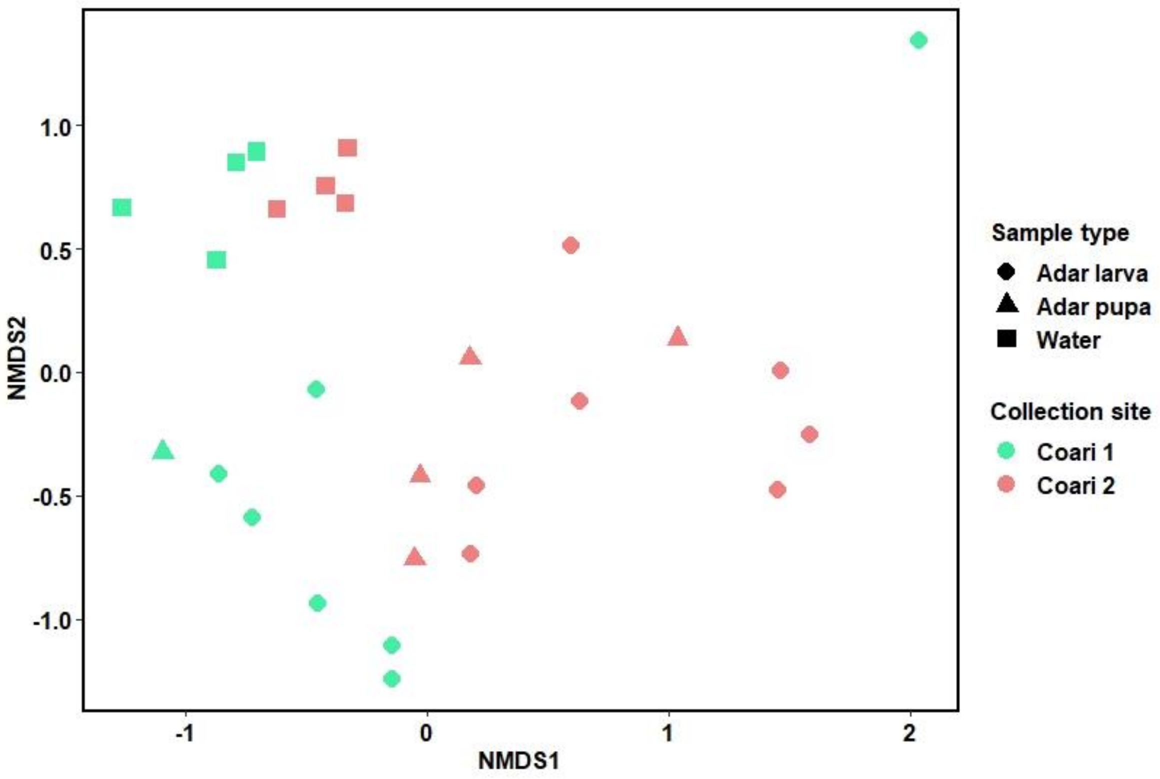

Fig. S4. Non-metric multidimensional scaling (NMDS) based on Bray-Curtis distances. The clustering patterns for each sample type (Adar larva, Adar pupa, and water) and collection site (Coari and Coari 2) are represented by a shape code and a color code, respectively.
